# Potential neuroprotective and anti-inflammatory effects provided by omega-3 (DHA) against Zika virus infection in human SH-SY5Y cells

**DOI:** 10.1038/s41598-019-56556-y

**Published:** 2019-12-27

**Authors:** Heloísa Antoniella Braz-De-Melo, Gabriel Pasquarelli-do-Nascimento, Rafael Corrêa, Raquel das Neves Almeida, Igor de Oliveira Santos, Paulo Sousa Prado, Victor Picolo, Andreza Fabro de Bem, Nathalia Pizato, Kelly Grace Magalhães

**Affiliations:** 1 Laboratory of Immunology and Inflammation, Department of Cell Biology, University of Brasília (UnB), 70910-900 Brasilia, Brazil; 2 Central Laboratory of Federal District (LACEN), 70830-010 Brasilia, Brazil; 3 Department of Physiological Sciences, University of Brasília (UnB), 70910-900 Brasilia, Brazil; 4 Department of Nutrition, University of Brasília (UnB), 70910-900 Brasilia, Brazil

**Keywords:** Viral infection, Inflammation

## Abstract

Zika virus (ZIKV) has a strong tropism for the nervous system and has been related to post-infection neurological syndromes. Once neuronal cells are infected, the virus is capable of modulating cell metabolism, leading to neurotoxicity and cellular death. The negative effect of ZIKV in neuron cells has been characterized. However, the description of molecules capable of reversing these cytotoxic effects is still under investigation. In this context, it has been largely demonstrated that docosahexaenoic acid (DHA), an omega-3 polyunsaturated fatty acid, is highly neuroprotective. Here, we hypothesized that DHA’s neuroprotective proprieties could have an influence on ZIKV-induced neurotoxicity in SH-SY5Y cells. Our data showed that pre-treatment of SH-SY5Y cells with DHA increased the cell viability and proliferation in ZIKV-infected cells. Moreover, DHA triggered an anti-inflammatory response in those infected cells. Besides, DHA was capable of restoring mitochondria function and number in ZIKV-infected SH-SY5Y cells. In addition, cells pre-treated with DHA prior to ZIKV infection presented a lower viral load at different times of infection. Taking together, these results demonstrated that DHA has a potential anti-inflammatory and neuroprotective effect against ZIKV infection in these neuron-like cells and could be a useful tool in the treatment against this virus.

## Introduction

First discovered in Uganda Forest in 1947, Zika virus (ZIKV) is an arthropod-born Flavivirus, transmitted by mosquitoes from *Aedes* genus, and, as recently discovered, an arbovirus sexually transmitted^1,2^. ZIKV has been associated with several neuronal alterations and congenital diseases^3^. Indeed, ZIKV is closely related to neurological disorders and presents a main tropism for nervous system, being isolated from animals born with microcephaly and infected adult mice brain^4,5^. Neuronal cells, both progenitors or differentiated ones, suffer a loss of homeostasis when infected with ZIKV and present considerable changes in cell metabolism during infection, due to the presence of specific required metabolites for viral replication^6,7^. ZIKV-induced neuronal alterations can directly impair neuronal homeostasis leading to decreased cellular proliferation and differentiation capacity of those cells, followed by cell death^7^.

It was also reported that negative influence of ZIKV in neuronal cells has a strong relationship with mitochondrial-sequestration of phospho-TBK1, an important factor that once relocated can cause a disruption in mitosis process, creating a critical environment to neuronal survival^8^. In addition, specific ZIKV proteins are capable of inhibiting Akt-mTOR pathway in neuronal stem cells, which plays essential role on neurogenesis process, cell maturation and migration^9,10^. Such mechanisms act synergistically to induce neuronal apoptotic cell death and loss of massive cell population during brain development and it can be accompanied by activation of inflammatory response^7,11,12^.

It is known that inflammation is a key process that orchestrates neuronal damage induced by ZIKV infection^13^. It has been reported that ZIKV intensively induces the generation of pro-inflammatory factors in microglia cells, such as IL-6 and MCP-1, when it infects human fetal brain^14^. In addition, pro-inflammatory response triggered by ZIKV in neuronal cells can be mediated by NLRP3 inflammasome activation, in a reactive oxygen species generation dependent manner, suggesting that oxidative stress plays an important role on ZIKV pathogenicity^15^. Besides, neurotoxic factors released by infected neurons are important to promote neuronal cell death during ZIKV infection^12^. Therefore, molecules with the ability to modulate inflammation could be useful to inhibit ZIKV pathogenicity. It has been demonstrated that Docosahexaenoic acid (DHA, C22:6(omega-3)), a polyunsaturated fatty acid derived from omega-3 family, can inhibit NLRP3 inflammasome^16,17^ and reduce intracellular reactive oxygen species^18^.

DHA is an essential fatty acid, therefore, it cannot be synthetized by *de novo* cell pathways and must be acquired by diet, mainly from cold water fish, or its oil intake, or from α-linoleic omega-3  fatty acid metabolism^19^. DHA has been described to be essential for normal function of diverse cell types of the organism, protecting against cardiovascular diseases and influencing positively retinal cells survival^20,21^. Moreover, the central impact of DHA in the organism can also be related to neuroprotection, increasing longevity of neuronal cells and decreasing neurodegeneration, inflammation and cognitive decline^22^.

Neurons and glial cells retain high levels of DHA in their cell membrane and this presence can positively influence electrochemical potential, membrane excitability, cell signaling and project an environment capable of maintaining cell integrity once homeostasis is threatened^23,24^. DHA also produces a potent lipid anti-inflammatory mediator called neuroprotectin-D1, a specialized pro-resolving mediator, which has anti-apoptotic actions, anti-oxidative properties, up-regulating the expression of proteins that induces cell survival, such as Bcl-2 and Bcl-xL^25^.

Here, we hypothesized that omega-3 (DHA) could protect against ZIKV-induced neurotoxicity. It is still unknown the effect of DHA supplementation during ZIKV infection in experimental neuronal-like models. Therefore, the aim of this work was to evaluate whether neuroprotective proprieties provide by DHA could have an influence against ZIKV infection in human SH-SY5Y cells.

## Results

### DHA protects against ZIKV-induced cytotoxicity

**Figure 1:**
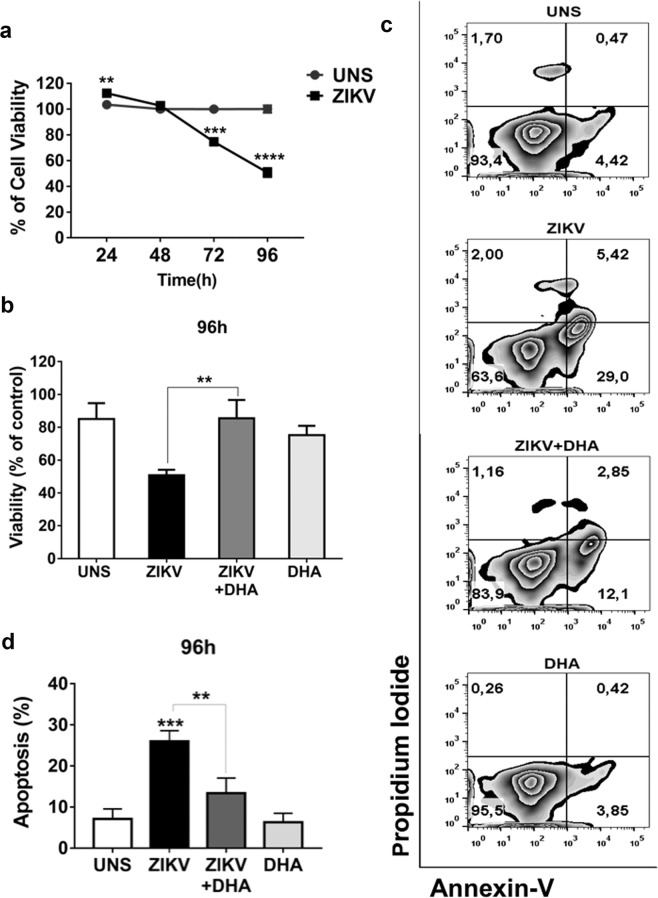
SH-SY5Y cells were infected with ZIKV (MOI 10). After 24, 48, 72 and 96 hours of infection cellular viability was assessed by 3-(4,5-Dimethyl-thiazol-2-yl)-2,5-diphenyl-tetrazolium bromide (MTT) assay and analyzed by spectrophotometry. (**a**) SH-SY5Y cells were pre-treated (or not) with DHA (12,5 μM) before ZIKV (MOI 10) infection. After 96 hours, cellular viability was assessed by MTT assay and analyzed by spectrophotometry. (**b**) All MTT graphs were made using GraphPad Prism version 6.00. Additionally, SH-SY5Y cells were treated (or not) with DHA (12,5 μM) before ZIKV infection (MOI 10) and after 96 hours apoptotic and lytic cellular death was evaluated by staining cells with Annexin V and Propidium Iodide, analyzed thereafter by flow cytometry. (**c**) The dot plot is representative of three independent experiments and was made using FlowJo Version 9. A quantitative graph of Annexin-V/PI assay using data from three independent experiments was also performed. (**d**) All experiments here were performed in quintuplicate, each being repeated at least three times independently. P values are represented by asterisks: p ≤ 0.01 (**), p ≤ 0.001 (***) and p ≤ 0.0001 (****).

We first analyzed the ability of omega-3 DHA to modulate cell viability during ZIKV infection in SH-SY5Y cells. Cell viability of human SH-SY5Y cells was analyzed by MTT assay in ZIKV-infected or uninfected cells after 24, 48, 72 and 96 hours of infection, in the presence or not of omega-3 DHA pre-treatment (Fig. 1a). We observed that ZIKV significantly reduced SH-SY5Y cells viability from 72 hours forward compared to uninfected cells. At 96 hours, ZIKV triggered a 50% loss of SH-SY5Y cells viability. Considering these results, we decided to investigate whether omega-3 DHA could protect SH-SY5Y cells cells from cell viability loss observed at 96 hours of infection. We verified that DHA greatly restored cell viability of the ZIKV-infected SH-SY5Y cells (Fig. 1b).

We also investigated what kind of induced cell death would be related to this reduction of cell viability caused by ZIKV. After 96 hours of infection, human SH-SY5Y cell death was evaluated and we observed that ZIKV triggered an increased apoptotic cell death compared to control cells (Fig. 1c). Apoptotic cell death was quantified (Fig. 1d) and DHA pre-treatment significantly decreased the number of apoptotic cells induced by ZIKV infection, restoring the number of viable cells (Fig. 1d).

### DHA enhances proliferative capacity of ZIKV infected cells

**Figure 2:**
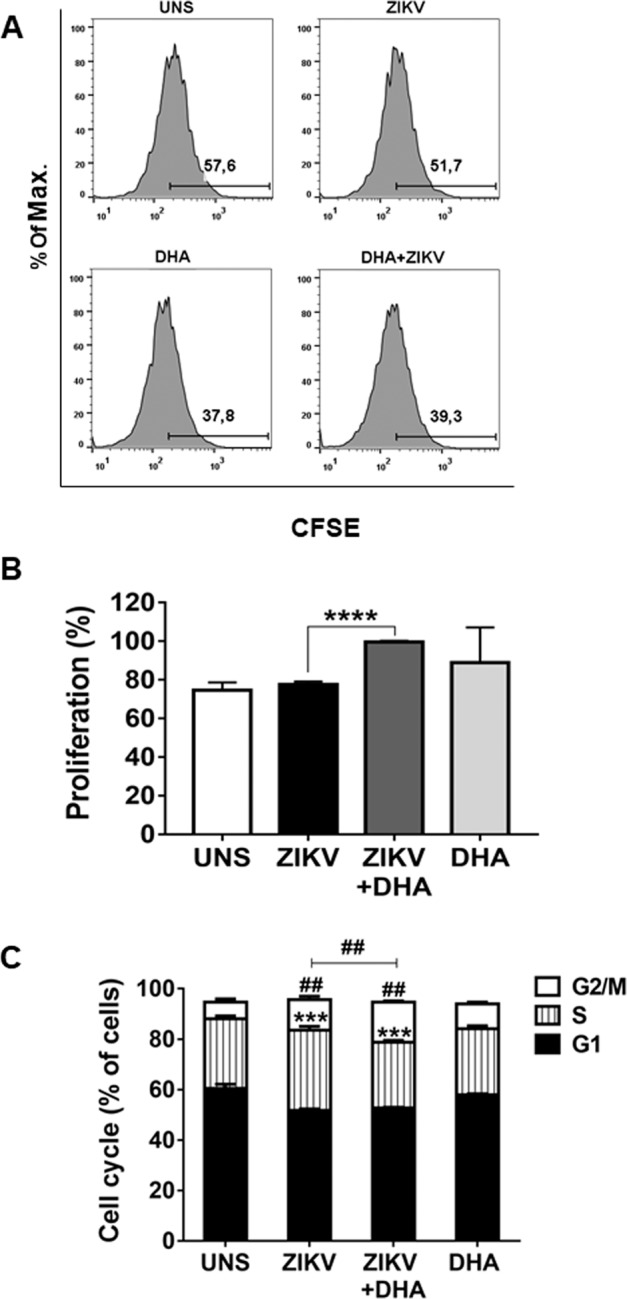
SH-SY5Y cells were pre-treated (or not) with DHA before ZIKV infection (MOI 10). After 96 hours, cell proliferation was assessed by Carboxyfluorescein Succinimidyl Ester (CFSE) staining and analyzed by flow cytometry. (**A**) Histograms are representatives of three independent experiments and were made using FlowJo Version 9. A quantitative graph of CFSE was also performed using data from three independent experiments. (**B**) Here, asterisks represent statistical significance with p < 0.001 (****). Additionally, SH-SY5Y cells were pre-treated (or not) with DHA (12,5 μM) before ZIKV infection (MOI 10) and after 72 hours, cell cycle was assessed by Propidium Iodide staining and analyzed by flow cytometry. (**C**) Flow cytometry data were plotted in the graph using GraphPad Prism. All experiments here were performed in triplicate, each being repeated at least three times independently. Here, symbols represent statistical significance comparing the untreated ZIKV infected cells with DHA-treated ZIKV infected cells in the G2/M phase (^##^p ≤ 0.001) or in the S phase (^***^p ≤ 0.01).

We analyzed the ability of omega-3 DHA to modulate cell proliferation during ZIKV infection in SH-SY5Y cells. We observed that ZIKV infection did not alter significantly the cell proliferation of the SH-SY5Y cells after 96 h of infection (Fig. 2A,B). However, DHA pre-treated cells prior to ZIKV infection presented a higher cell proliferation activity compared with infected cells in the absence of DHA (Fig. 2A,B). This result was further confirmed when we analyzed cell cycle of these cells under similar experimental condition. ZIKV infection did not modify significantly the percentage of cells in G2/M phase when compared with uninfected cells after 72 h of infection (Fig. 2C). Moreover, DHA pre-treated cells prior to ZIKV infection presented a significantly higher percentage of cells in G2/M phase (Fig. 2C), indicating that omega-3 DHA has the ability to enhance cell proliferation ability of ZIKV-infected SH-SY5Y cells.

### DHA softens pro-inflammatory response induced by ZIKV infection

**Figure 3:**
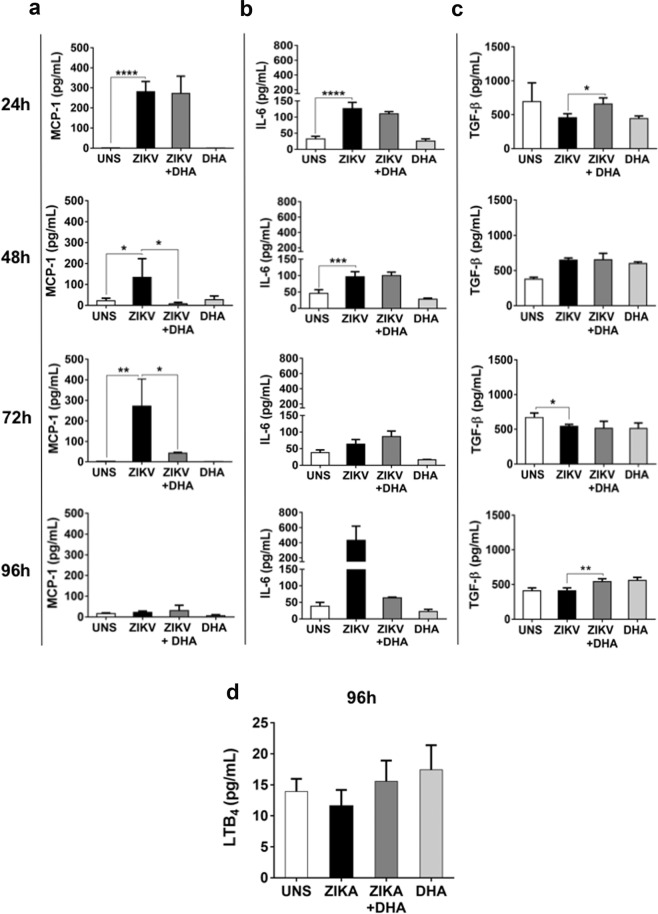
SH-SY5Y cells were pre-treated (or not) with DHA (12,5 μM) before ZIKV infection (MOI 10) and after 24, 48, 72 and 96 hours, inflammatory cytokines and chemokines released in the supernatant as MCP-1 (**a**), IL-6 (**b**), and TGF-β (**c**), was assessed by ELISA. Lipid inflammatory mediator Leukotriene B_4_ was also assessed in the supernatant only at 96 hours of infection. (**e**) All the experiments above mentioned were analyzed by spectrophotometry and graphs were made using GraphPad Prism Version 6.00. Here, asterisks represent statistical significance with p ≤ 0.05 (*) and p ≤ 0.01 (**). All experiments here were performed in quintuplicate, each being repeated at least three times independently.

We analyzed whether ZIKV infection could modulate the secretion of cytokines, chemokines and lipid mediators by SH-SY5Y cells, after 24, 48, 72 and 96 hours of infection. We observed that ZIKV infection induced a pro-inflammatory profile in SH-SY5Y cells by triggering the secretion of higher levels of the pro-inflammatory cytokine IL-6 and the chemokine MCP-1, since initial times of infection (24 h) (Fig. 3a,b). DHA pre-treatment was capable of reducing this secretion at 48 and 72 hours for MCP-1 and at 96 hours of infection for IL-6, where this cytokine had its apex of secretion compared to other times.

It was also analyzed an anti-inflammatory cytokine during ZIKV infection and the role of omega-3 DHA in this process. For this, we analyzed TGF-β secretion in ZIKV-infected SH-SY5Y cells after 24, 48, 72 and 96 hours of infection. We observed that ZIKV decreased TGF-β secretion at 72 hours of infection (Fig. 3c). Moreover, DHA pre-treatment was capable of increasing TGF-β secretion levels at 24 and 96 hours of infection in these cells. IL-10 was also measured, but no significant differences were observed in all conditions and time points analyzed (data not shown).

We also investigated whether ZIKV could modulate the secretion of a lipid inflammatory mediator, the Leukotriene B_4_ (LTB_4_) and the role of omega-3 DHA in this process. We observed that ZIKV did not have any influence in LTB_4_ secretion SH-SY5Y cells after 96 hours of infection. Similarly, DHA pre-treatment prior to ZIKV infection did not significantly modified LTB_4_ levels (Fig. 3d). Taking together, these results demonstrated that DHA pre-treatment has a potential to soften exacerbated inflammatory profile induced by ZIKV, not only by decreasing pro-inflammatory factors, but also increasing an anti-inflammatory cytokine. Although DHA pre-treatment decreased pro-inflammatory cytokine levels, it is important to emphasize that it does not abrogate the secretion of these mediators by the pre-treated cells, allowing an important factor for the activation of immune response against ZIKV infection.

### DHA prevents the mitochondrial dysfunction and reactive species production induced by ZIKV

**Figure 4:**
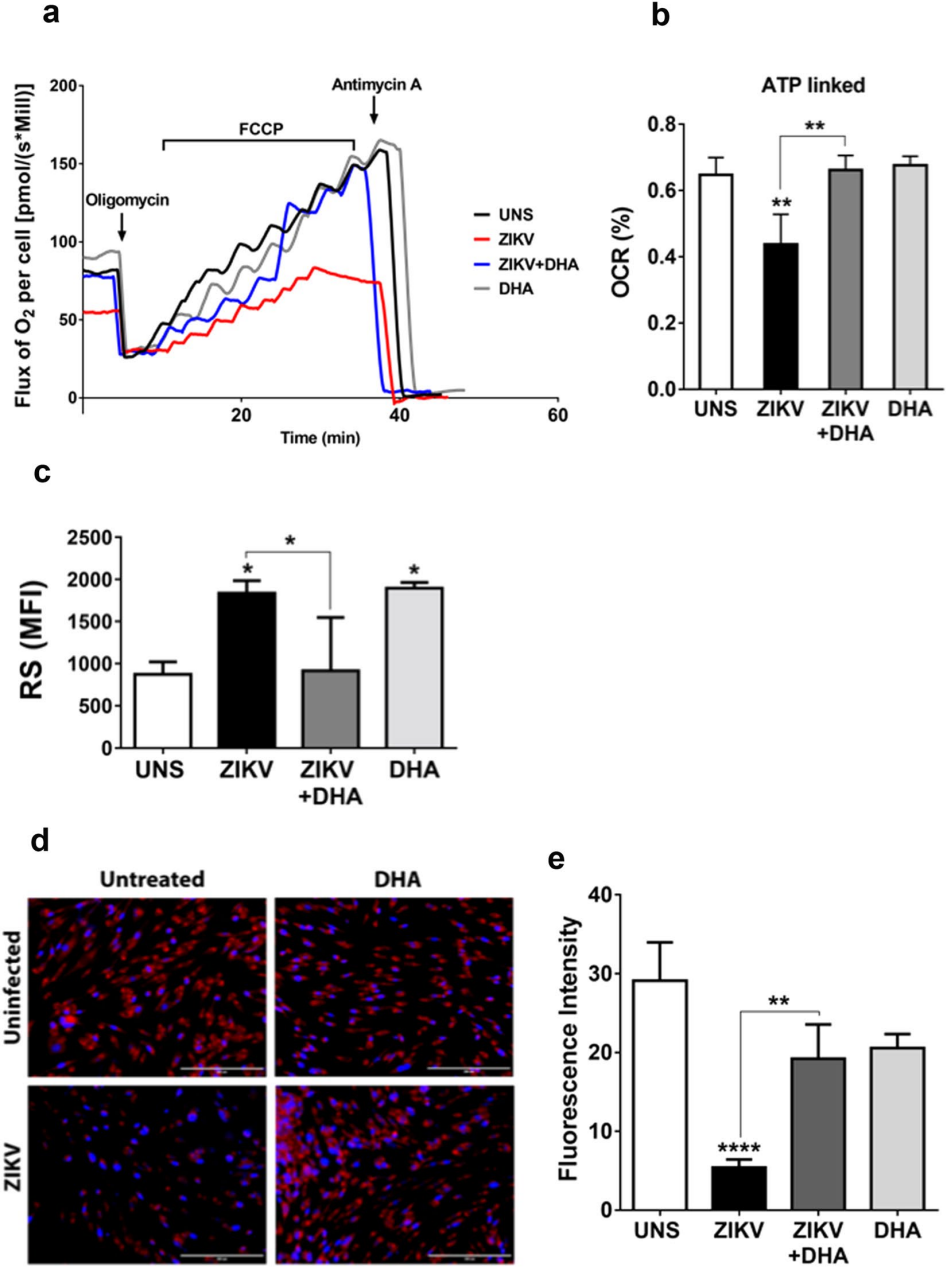
SH-SY5Y cells were pre-treated with DHA (12,5 µM) before ZIKV infection (MOI 10) and after 48 hours, oxygen consumption rate (OCR) (**a**) and ATP-linked (**b**) was evaluated with a high respirometry assay under different inhibitors action, as oligomycin (1,25 µM), FCCP (100 nM) and antimicyn-A (2,5 µM). Graphs were made using GraphPad Prism Version 6.00. SH-SY5Y cells were pre-treated (or not) with DHA (12,5 µM) before ZIKV infection (MOI 10) and after 96 hours, reactive species was assessed by 2′,7′-Dichlorofluorescin diacetate (DCF-DA) and analyzed by flow cytometry. (**c**) Graphs were made using GraphPad Prism Version 6.00. This experiment was performed in quintuplicate, each being repeated at least three times independently. Additionally, SH-SY5Y cells were pre-treated (or not) with DHA (12,5 µM) before ZIKV infection (MOI 10) and live mitochondria were assessed by MitoTracker staining and analyzed by fluorescence microscopy. (**d**) Fluorescence intensity obtained in the independent experiments was quantified using ImageJ. (**e**) This experiment was performed in triplicate, each being repeated at least three times independently. Asterisks represent statistical significance with p ≤ 0.01 (**), p ≤ 0.001 (***) and p ≤ 0.0001 (****).

Getting deeper into the mechanisms through which ZIKV induced SH-SY5 cell death, cellular oxygen consumption was evaluated through a high resolution respirometry assay. The mitochondrial function of SH-SY5Y cells was determined after 48 hours after ZIKV infection, a period of time where cell viability was not affected by the virus (Fig. 4a,b). We observed that mitochondrial dysfunction was induced by ZIKV SH-SY5Y cells, even before cell death had been noticed. Routine respiration was lower in ZIKV-infected cells in the absence of the DHA pre-treatment compared with untreated or uninfected cells. In addition, oxygen consumption directed to ATP synthesis (ATP-linked) was lower on ZIKV-infected cells compared to uninfected cells, suggesting that mitochondrial damage by ZIKV can start at initial times of infection. DHA also restored ATP-linked oxygen consumption, protecting against SH-SY5Y cells mitochondrial dysfunction triggered by ZIKV infection.

Taking to account that ZIKV-mediated cell damage could be related to oxidative stress, we investigated whether DHA can protect SH-SY5Y cells from ZIKV-induced reactive species generation. At initial times (3 and 24 hours), reactive species generation was not modulated (data not shown). After 96 hours of infection (Fig. 4c), considered a late time of infection, ZIKV significantly increased reactive species generation compared to uninfected cells. However DHA pre-treatment was capable of preventing reactive species generation in ZIKV-infected cells.

Then, we addressed whether ZIKV could impair the number of viable mitochondria in SH-SY5Y cells and whether omega-3 DHA could protect cells from this damage. For this, MitoTracker staining was performed in SH-SY5Y cells and analyzed by fluorescence microscopy. It was possible to observe that ZIKV infected cells presented the lowest amount of live cells and a significant reduction of viable mitochondria compared to all other conditions analyzed (Fig. 4d). Moreover, DHA pre-treatment prior to ZIKV infection infection was capable of protecting against ZIKV-induced loss of mitochondria viability (Fig. 4d,e), corroborating with MTT assay (Fig. 1b).

### DHA reduces ZIKV load and cytopathic effect in infected cells

**Figure 5:**
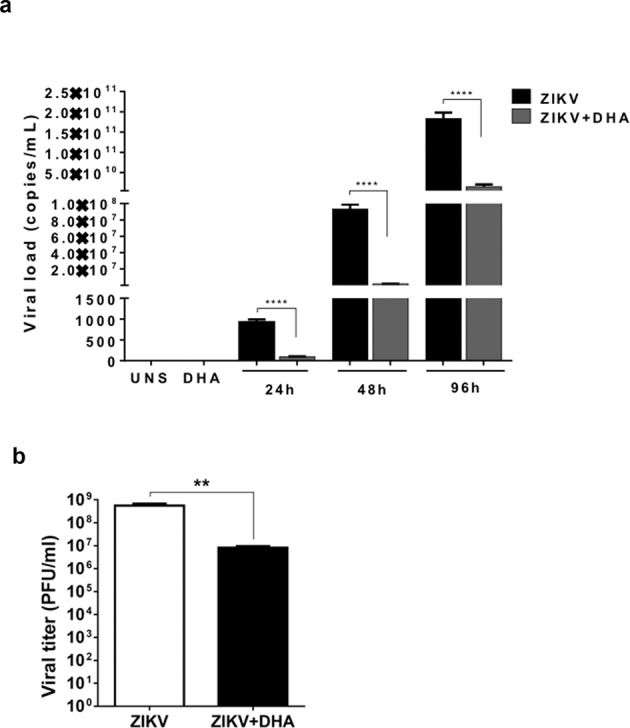
SH-SY5Y cells were pre-treated (or not) with DHA (12,5 µM) and after 24, 48 and 96 hours of infection, intracellular viral load was assessed by RNA extraction and analyzed by RT-qPCR. (**a**) This experiment was performed in triplicate. Additionally, SH-SY5Y cells pre-treated (or not) with DHA (12,5 µM) and after 96 hours of ZIKV infection, viral titers quantification was assessed by TCID-50 (50% Tissue culture Infective Dose), followed by further mathematical conversion to PFU. (**b**) This experiment was performed in sextuplicate. Asterisks represent statistical significance with p ≤ 0.01 (**) and p ≤ 0.0001 (****).

Finally, we addressed whether changes in mitochondrial function and other intracellular parameters by omega-3 could have an impact on viral load in SH-SY5Y cells infected with ZIKV. For this, we analyzed the viral burden in DHA pre-treated or untreated SH-SY5Y cells infected with ZIKV. The results showed that ZIKV is significantly increasing its charge over times of infection, getting at 10^11^ viral copies/mL at 96 hours. Surprisingly, when SH-SY5Y cells were pre-treated with DHA prior to ZIKV exposure, the viral load significantly decreased in all times tested (p < 0,01), since 24 hours of infection. This suggests that cellular modulation exerted by DHA in ZIKV-infected SH-SY5Y cells may occur since early periods and may play an important role in the reduction of viral particles that could trigger cell death observed at later periods of infection. In addition, we also investigated whether omega-3 DHA could have an impact on the cytopathic effect triggered by ZIKV in SH-SY5Y cells by performing a PFU assay after 96 hours of infection (Fig. 5b). Here, we observed that ZIKV triggered cytopathic effects in SH-SY5Y cells, and omega-3 DHA may have a protective effect in this process, since cells pre-treated with DHA prior to ZIKV infection presented a diminished number of plaque-forming units compared to untreated cells infected with ZIKV (Fig. 5b).

## Discussion

Taking into account the scenario of neuronal alterations caused by ZIKV, this paper explored neuroprotective properties derived from the omega-3 polyunsaturated fatty acid (PUFA) docosahexaenoic acid (DHA). This PUFA is widely studied as a bioactive anti-inflammatory nutrient and is also an important component of neuronal membrane, playing key roles in intracellular mechanisms that leads to neuronal survival on a stressful environment^19,22,26^. Despite the fact of both ZIKV and DHA effects on neuronal cells have already been elucidated, there is nothing exploring how they could influence each other, once ZIKV and DHA have opposite effects on the same cell type^12,22^. For this reason, the present study is a pioneer in the neuroprotection field provided by DHA against ZIKV infection, and, at the same time, appends to the scarce literature about this fatty acid effects on diverse infections^27,28^.

SH-SY5Y cells are widely used as an important model for approaches focused on neuronal cells physiology and neurological diseases and several studies have provided very pertinent biological and pharmacological findings using SH-SY5Y as a neuronal-like *in vitro* model^29–34^. In our work, we could observe that ZIKV decreased SH-SY5Y cells viability in a time-dependent manner, corroborating with other studies that uses the same cell line^31,33^. At initial times, as 24 and 48 hours of infection, ZIKV maintained cell viability around 100%. However, despite there is no cell death induction at these times, ZIKV caused intracellular alterations that will be essential for triggering SH-SY5Y cells loss of viability at later times of infection, specifically from 72 hours^12^. In this context, DHA neuroprotective properties were tested in a pre-treatment prior to ZIKV infection for 96 hours. This time point was chosen due to the massive cell viability loss observed at that moment. Even at this harmful environment caused by four days of ZIKV infection, we found that DHA could restore a major percentage of cell viability, compared to untreated cells. This counter-effect could be better analyzed when we looked for more specific mechanisms involved in the SH-SY5Y cell death caused by ZIKV. Indeed, ZIKV-induced apoptotic cell death has already been reported^4,7,11^. Here, we demonstrated that DHA played an important role on protecting more than a half of the cell population. It was expected since omega-3 fatty acids are capable of preventing neuronal apoptosis under different conditions^35–37^.

We also looked for any modulation on cell cycle and proliferative capacity on SH-SY5Y cells. For this, we analyzed cell proliferation by CFSE assay and cell cycle looking at the phases related to mitosis process, known as G2/M phases. It has been demonstrated that ZIKV can decrease cell proliferation of the neuronal progenitor cells^6,8^. Here we demonstrated that ZIKV did not modify SH-SY5Y cell proliferation, neither G2/M phase of the cell cycle progression in SH-SY5Y cells. Therefore, the loss of SH-SY5Y cells viability observed in previous experiments was related to ZIKV-induced cell death and not by any modulation of proliferative capacity of the immortalized neuronal cell. Nevertheless, DHA pre-treatment prior to ZIKV infection increased the percentage of ZIKV infected cells proliferating (on G2/M phase) compared to untreated cells. Indeed, PUFAs have the ability of stimulating intracellular pathways related to neuronal proliferation^19,38^.

Another important field to explore is the modulation of inflammatory response to ZIKV infection and the role of omega-3 DHA in this process SH-SY5Y cells^12,19^. We observed that ZIKV increased the secretion of pro-inflammatory components, especially IL-6 and MCP-1 since initial times of infection, in agreement with a previous work that described an inflammatory profile of astrocyte and microglia during ZIKV infection^14,39^. On the other hand, DHA pre-treatment was capable of softening pro-inflammatory components induced by ZIKV in addition to increasing secretion of anti-inflammatory cytokine TGF-β. It is important to emphasize that inflammatory profile plays a relevant function during ZIKV pathogenesis in different cell types^40^, but, at the same time, immune system activation is essential for combating the infection^41,42^. A balance between anti- and pro-inflammatory agents is the key for homeostasis restoration. In this context, DHA reduced significantly inflammatory markers in ZIKV-infected SH-SY5Y cells, but it maintained the secretion of these molecules at a sufficient level for activating the immune response to eliminate the infection. Checking other inflammatory mediators, it was also explored whether ZIKV could modulate the secretion of a lipid inflammatory mediator, the Leukotriene B_4_ (LTB_4_), and the role of omega-3 DHA in this process. We observed that ZIKV did not have any influence in LTB_4_ secretion in SH-SY5Y cells after 96 hours of infection. As already described^43^, DHA can be retroconverted in eicosapentaenoic acid (EPA), a 20 carbon omega-3 fatty acid family^44^. EPA produces Leukotrien B5 (LTB_5_), which is less potent eicosanoid mediator than LTB_4_, originated from arachidonic acid, an n-6 family fatty acid^45^. DHA treatment could enhance LTB_5_ production modulating LTB_4_ effects on SH-SY5Y cells. However, no differences on LTB_4_ secretion were observed among all conditions analyzed here.

ZIKV-induced mitochondria stress was previously reported in neuronal stem cells. This study showed that ZIKV infection causes mitochondrial apoptosis in neuronal stem cells via mitochondrial sequestration of phospho-TBK1 during mitosis^8^. Our results showed that ZIKV caused mitochondrial dysfunction in SH-SY5Y cells as early as 48 hours of infection, therefore earlier than cell death induction (that occurred after 72 hours). High resolution respirometry analysis showed a significant reduction on ATP-linked oxygen consumption rate in ZIKV-infected SH-SY5Y cells. Differently from neuronal stem cells that mostly rely on the glycolysis form energy production, mature neurons depend on OXPHOS to sustain its high metabolic rate^46^. In this sense, we may suggest that ZIKV modulates the cellular metabolism in SH-SY5Y cells, impairing the energy demand and making these cells particularly vulnerable to oxidative stress and apoptosis. In fact, we verified that mitochondrial dysfunction at the early stage of ZIKV infection later culminated in effective cell death^47^. For concluding mitochondrial alterations in this context, we looked for amount of viable mitochondria and we noticed that there was a considerable reduction on viable mitochondria on ZIKV infected cells. By contrast, the pre-treatment of SH-SY5Y cells with omega-3 DHA turned cells more efficient to resist to ZIKV-induced metabolic changes. DHA efficiently restored the mitochondrial dysfunction induced by ZIKV and preserved the mitochondrial viability after 72 hours of ZIKV infection in SH-SY5Y cells.

At later times of ZIKV infection, where inflammatory profile is harmful and cell death processes are being triggered, ZIKV infected cells presented higher levels of reactive species. In the other hand, DHA pre-treated cells prior to ZIKV infection presented generation of the reactive species in levels very similar to uninfected cells. Reactive species are important factors on triggering cellular damage and present a role on activation of NLRP3 inflammasome during ZIKV infection^15^. Reactive species generation reduction induced by omega-3 DHA in ZIKV-infected SH-SY5Y cells, suggests a protection property of DHA against cellular damage and it could trigger inhibition of the NLRP3 inflammasome activation, as an anti-inflammatory mechanism.

After exploring a couple of effects that are modulated by ZIKV, we asked whether DHA responses could influence on intracellular viral load throughout different times of infection. We observed that viral load was significantly decreased in the presence of DHA, since initial times (24 hours). We also quantified infective virus particle by a cytotoxicity assay, which also demonstrated that DHA was capable of reducing ZIKV particles that could induce SH-SY5Y cell death.

As conclusion, we showed that ZIKV infection of SH-SY5Y cells induced reactive species production and mitochondrial dysfunction. These alterations in mitochondrial function can be suggested as an important factor for increased apoptotic events triggered by ZIKV in SH-SY5Y cells. In contrast, DHA pre-treatment of SH-SY5Y cells inhibited RS production and restored mitochondria functionality that was disturbed by ZIKV infection. Consequently, apoptosis was inhibited in those cells and cell viability was maintained. DHA pre-treatment softens secretion of pro-inflammatory cytokines and increase the secretion of anti-inflammatory TGF-β. This work also showed that DHA stimulation was capable of decreasing viral load in infected cells, suggesting that DHA responses on SH-SY5Y cells protects from ZIKV-induced neurotoxicity and interfered on viral load production in those cells.

Therefore, our results suggest that omega-3 DHA can induce alterations on SH-SY5Y cells that may keep them more resistant against ZIKV infection by: (I) reducing cell death and increasing cell viability; (II) enhancing cell proliferation; (III) softening pro-inflammatory response; (IV) preventing mitochondrial dysfunction and reactive species production; and (V) reducing viral load and virus infectivity. This effect is not just preventing ZIKV neurotoxicity, but also interfering on its own viral load production and their infectivity. The decrease of viral particles can dramatically impact the effects observed on ZIKV pathogenesis. Hence, our findings revealed that the omega-3 DHA has a neuroprotective effect on neuronal-like cells SH-SY5Y against ZIKV infection.Taking together, our work presents important mechanisms that may be involved in this neuroprotective function of this PUFA against ZIKV infection and confirms the potential of novel applications of omega-3 fatty acids as protective agents against viral infection that could impact neuronal tissue.

## Methods

### Cell line and virus stocks

Human neuroblastoma cells (ATCC SH-SY5Y) were cultured in Dulbecco’s Modified Essential (DMEM) and F12 Medium, supplemented with 2% of fetal bovine serum and 100 µM of Penicilin-Streptomicin (Sigma Aldrich) and maintained at 37 °C and 5% of CO_2_. All the experiments bellow were performed with SH-SY5Y cell line, the model of human neuronal-like lineage. For virus expansion process, it was used C6/36 mosquito cell line cultured in TC-100 medium and supplemented with 2% of fetal bovine serum, both acquired from Sigma Aldrich, maintained at 37 °C in absence of CO_2_. Vero cell line (ATCC CCL-81) was also used for viral expansion, cultured in DMEM and supplemented with 10% of fetal bovine serum. ZIKV_PE243_ (gene bank reference number KX197192) was kindly provided by Dr. Rafael Freitas de Oliveira França (FIOCRUZ, PE, Brazil) after isolation in 2015 from a human case that occurred in the state of Pernambuco (Brazil). The virus was propagated in C6/36 and, after one passage, propagated as well in Vero cells. Stocks were aliquoted and frozen at −80 °C, where each vial was used a single time. Viral titers were determined by plaque-forming unit (PFU) and confirmed by RT-PCR.

### Pre-treatment and Infection

SH-SY5Y cells were pre-treated with docosahexaenoic acid (DHA–Sigma-Aldrich) on a concentration of 12.5 µM and incubated for one hour. After treatment, cells were once washed with Phosphate Buffered Saline (PBS) and infected thereafter. As a control, cells were incubated with DMEM 2% in absence of DHA. SH-SY5Y cells, pre-treated and non-treated, were infected with ZIKV_PE243_ (MOI 10) and were incubated for 2 hours in 37 °C and 5% of CO_2_. After this time of infection, DMEM supplemented with 2% of fetal bovine serum was added and the cells remained in the incubator during 24, 48, 72 and 96 hours, according to the analysis. Uninfected cells were used as a negative control.

### Cell viability by MTT Assay

SH-SY5Y cells were plated in 96-well plates (n = 4) and pre-treated (or not) with DHA and infected (or not) after treatment with ZIKV. After 24, 48, 72 and 96 hours of infection, cell viability dependent on mitochondrial activity was evaluated by 3-(4,5-Dimethyl-thiazol-2-yl)-2,5-diphenyl-tetrazolium bromide (MTT Sigma-Aldrich) in a concentration of 10% (stocks concentration at 5 mg/ml diluted in Phosphate Buffered Saline). The plates were incubated at 37 °C and 5% of CO_2_ for 1 hour and then analyzed by spectrophotometry at 570 nm.

### Cell death analysis by Annexin V and PI

SH-SY5Y cells were plated in 24-well plates pre-treated with DHA and posteriorly infected with ZIKV (as control untreated and/or uninfected cells), and after 96 hours of infection, cell death was analyzed by flow cytometry (FACSVerse) and using FITC Annexin V Apoptosis detection Kit I (BD Biosciences) and Propidium Iodide (Sigma-Aldrich). This experiment was performed in triplicate, each being repeated at least three times independently.

### Proliferation analysis by CFSE

SH-SY5Y cells were plated in 24-well plates and were marked with Carboxyfluoroscein Succinimidyl Ester (CFSE) (Thermo Fisher), in a concentration of 5 µM. Cells were incubated with CFSE for fifteen minutes protected from light at room temperature, then were blocked with DMEM and F12 supplemented 10% of fetal bovine serum. Pre-treatment (or non-treatment) and infections (as control uninfected cells) were made thereafter and cells were incubated, protected from light, for 96 hours. Proliferative capacity was analyzed by Flow Cytometry, using a FACSVerse, after incubation. This experiment was performed in triplicate, each being repeated at least three times independently.

### Cell-cycle analysis by PI

In order to evaluate the impact of pre-treatments on SH-SY5Y cell cycle, cells were seeded in 24 well plates (n = 3) and exposed to ZIKV or DHA or both. After 72 hours of incubation, cells were detached and fixed using ethanol 70% (v/v) for 2 hours. Cells were then resuspended in 50 µL of PI solution [0.1% sodium citrate (v/v), 0.1% Triton-X (v/v), 20 µg/mL propidium iodide, 50 µg/mL RNAse, phosphate buffer saline (PBS)] at pH 7.4 and incubated for 30 min at room temperature. Cells were diluted in PBS and analyzed using a FACSVerse flow cytometry (Becton & Dickenson, USA) using linear scale. A total of 10,000 events were obtained per sample. This experiment was performed in triplicate, each being repeated at least three times independently.

### Inflammatory profile by ELISA

After 24, 48, 72 and 96 hours of infection, secretion of the cytokines IL-6, IFN-γ and TGF-β and the chemokine MCP-1 was analyzed by Enzyme-Linked Immunosorbent Assay (ELISA) following the recommendations of manufacturer (BD Biosciences) and analyzed by spectrophotometry at 450 nm. This experiment was performed in quintuplicate, each being repeated at least three times independently.

### Leukotriene B_4_ dosage

After 96 hours of infection, SH-SY5Y cells supernatant was used for leukotriene B_4_ measurement. The experiment was made using Cayman Chemical Leukotriene B_4_ ELISA kit following the recommendations of manufacturer. This experiment was performed in quintuplicate, each being repeated at least three times independently.

### Intracellular reactive species analysis

SH-SY5Y cells were plated in a 24-well plate, pre-treated with DHA and posteriorly infected with ZIKV, with uninfected and/or untreated cells as control. 96 hours after infection and treatments, intracellular reactive species (RS) were stained with 2′,7′-Dichlorofluorescin diacetate (DCF-DA) (Sigma-Aldrich), in a concentration of 20 µM. The staining was done with cells added to the plate for minimizing stresses that occur during cell detaching process and cells were incubated for 30 minutes at culture conditions, followed by serial washes and fixation. After fixation with Formalin (3.7%), cells were suspended and RS generation was measured using a FACSVerse flow cytometry (Becton & Dickenson, USA). This experiment was performed in triplicate, each being repeated at least three times independently.

### High-resolution respirometry

To evaluate the mitochondrial oxygen consumption, SH-SY5Y cells were plated in a 24-well plate, DHA pre-treated (or not) and infected with ZIKV, using as control uninfected cells. After 48 hours of infection, approximately 10^6^ cells were suspended in DMEM and F12 in the absence bovine fetal serum. Oxygen consumption rate (OCR) was measured using a high-resolution respirometer (OROBOROS Oxygraph-2k) at 37 °C, continuous stirring at 750 rpm and a final volume of 2 mL. For calibration of the equipment, in each experiment day was used DMEM and F12, the same medium used for cell culture. The basal OCR was calculated upon stabilization of oxygen flux and after cell the addition. Different substrates were added to the chamber containing cells and oxygen consumption measured after stabilization of the signal. As substrates, were used Olygomicin 1.25 µM, from which ATP-linked OCR could be obtained in the final analysis; Carbonyl cyanide-p-trifluoromethoxyphen-ylhydrazone (FCCP) in pulses of 100 nM, in order to analyze maximum respiratory capacity; At last, respiration was inhibited by Antimicin-A 2.5 µM, in order to analyze residual oxygen consumption (ROX). The ATP-linked OCR was presented as the difference between the OCR before (basal respiration) and after (proton leaking) addiction of Olygomicin, normalized by the basal respiration. This experiment was performed in quintuplicate, each being repeated at least three times independently.

### Mitochondrial detection by microscopy

SH-SY5Y cells were plated on a coverslip, in a 24-well plate, pre-treated with DHA and posteriorly infected with ZIKV, with uninfected and/or untreated cells as control. After 72 hours of infection and treatments, mitochondria of living cells were marked by MitoTracker Red CMXRos (Thermo Fisher), in a concentration of 100 nM. The reagent was diluted in DMEM and F12 medium without fetal bovine serum and cells were exposed to MitoTracker for 45 minutes. After staining, cells were vigorously washed with PBS and then fixed for 15 minutes with Formalin (3.7%). After fixation, cells were washed again and had their nucleus marked with 4′,6′-diamino-2-fenil-indol (DAPI) for 5 minutes, in a DAPI/PBS ratio of 1:5000. After washes, samples were analyzed on EVOS Cell Imaging Systems fluorescence microscope (Thermo Fisher). Fluorescence quantification was performed using ImageJ software. This experiment was performed in triplicate, each being repeated at least three times independently.

### Viral load by RT-PCR

To monitor viral loads, RT-PCR was conducted after RNA extraction of SH-SY5Y cells treated (or not) and infected with ZIKV (or not). RNA extraction (as described previously by Prado *et. al*.^48^) was made by High Pure Viral Nucleic Acid Version 18 Kit (Roche Diagnosis), with a single step reaction provided by LightCycler Multiplex RNA Virus Master Version 03 (Roche Diagnosis). RNA standards, in a final volume of 20 µl, were reverse transcribed; Primer concentration was 0.5 µM and probe was used in a concentration of 200 nM. A standard curve, with a serial dilution of known titration virus, was also made by RT-PCR to calculate viral loads per mL. This experiment was performed in triplicate, each being repeated at least three times independently.

### Viral titers protocol- TC1D_50_ assay

SH-SY5Y was evaluated in two conditions: DHA-treated and non-treated ZIKV-infected cells and analyzed for TCID50 (50% Tissue Culture Infective Dose). For endpoint TCID_50_ determination 60000 cells were plated and grown into 96 wells plate. They were inoculated thereafter with 10 -fold dilution of ZIKV samples. The endpoint titration resulted in final ZIKV dilutions of 10^−1^–10 ^−9^ fold on the cells in n = 6. Cells were incubated for 4–5 days at 37 °C and monitored to ZIKV- induced cytopathic effect and plaque formation. TCID50/mL was calculated according to Reed and Muench method^49^. After TCDI50 obtained values, the PFU/ml was predicted using Poisson distribution, which multiplies the TCID_50_ titer (per ml) by 0.7 to predict the mean number of PFU/ml.

### Statistical analysis

All experiments here were performed at least in triplicate, each being repeated at least three times independently. Mean ± standard deviation (SD) are shown for the number of replicates of each experiment (n ≥ 3), those are specified in *Figures Legend*. For flow cytometry graphs, mean ± standard deviation represents the merging of the obtained data of three independent experiments. Statistical analysis was made using GraphPad Prism (6.00) and the statistical test for multiple comparisons were made with one-way ANOVA and the Tukey post-test. P values are represented by asterisks: p ≤ 0.05 (*), p ≤ 0.01 (**), p ≤ 0.001 (***) and p ≤ 0.0001 (****).
